# Preparation and characterization of liquefied eggplant branch bio-based controlled-release fertilizer

**DOI:** 10.1186/s13065-024-01180-9

**Published:** 2024-04-12

**Authors:** Yanle Guo, Fengyuan Zhuang, Qunxiang Cui, Shugang Zhang, Zhenping Hao, Yiyun Shi, Hao Lu, Xiaoqing Shi

**Affiliations:** 1 College of Horticulture and Landscape Architecture, Jinling Institute of Technology, Nanjing, 210038 China; 2 National Engineering Laboratory for Efficient Utilization of Soil and Fertilizer Resources, National Engineering and Technology Research Center for Slow and Controlled Release Fertilizers, College of Resources and Environment, Shandong Agricultural University, Tai’an, 271018 China; 3 Key Laboratory of Crop Genetics and Physiology of Jiangsu Province, Co-Innovation Center for Modern Production Technology of Grain Crops of Jiangsu Province, Yangzhou University, Yangzhou, 225009 China; 4 Huacheng Vegetable Cooperative Co., Ltd, Nanjing, 211299 China

**Keywords:** Coated material, Eggplant branch, Organosilicon, Hydrophobic, Characteristics

## Abstract

Bio-based coating materials have received increased attention because of their low-cost, environmentally friendly, and sustainable properties. In this paper, a novel coating material was developed to coat ureas using bio-based coating material derived from liquefied eggplant branches to form controlled-release ureas (CRUs). Also, the optimum proportion of liquefier was studied. Furthermore, dimethyl siloxane was used to modify liquified eggplant branches to make them hydrophobic, resulting in hydrophobic controlled-release ureas (SCRUs). This hydrophobic-enabled coating is environmentally friendly and highly efficient. The products were characterized by specific scanning electron microscopy, energy-dispersive X-ray spectroscopy, Fourier transform infrared spectroscopy, thermogravimetric analysis, and differential scanning calorimetry, and the water contact angles of CRUs and SCRUs were determined. The nutrient-release characteristics of the SCRUs in water were determined at 25 °C and compared with those of CRUs. The results showed that the modification with dimethyl siloxane reduced the N release rate and increased the longevity of the fertilizer coated with hydrophobic bio-based coating material. In addition, organosilicon atoms on the SCRU surface also block the micro-holes on the coating and thus reduce the entry of water onto the coating. The results suggest that the new coating technology can create a hydrophobic surface on bio-based coating material and thus improve their controlled-release characteristics.

## Introduction

The world’s population has now reached 8 billion and will approach 9.7 billion by 2050 [1], requiring a corresponding increase in crop production and food supply. Agricultural productivity needs to be raised accordingly, which requires the effective utilization of fertilizer [2]. However, the low efficiency of traditional fertilizers has become a common problem in the application of chemical fertilizers [3]. Compared with traditional fertilizers, coated controlled-release ureas have the advantages of diversified coating materials, good controlled-release performance, and a simple production process [4, 5]. The principle behind the use of the coating is to form a protective film on the surface of instant fertilizer particles to block water and delay nutrient release [6, 7]. However, traditional coated fertilizers have problems, such as strong dependence on petrochemical resources of resin film materials, high prices, and long degradation cycle polluting soil [8–10]. For example, the membrane shell of controlled-release fertilizer prepared by inorganic coated materials is brittle [11, 12], which limits its wide application in field crops; polymer coating materials, such as polyolefin, polyvinyl chloride, and acrylic resin [13, 14], are obtained from petrochemical resources [15]. The cost of polymer materials is also increasing with the increasing shortage of petrochemical resources and the global trend of reducing carbon emissions [14, 15]. Moreover, most polymer materials have the disadvantage of not being biodegradable, resulting in environmental pollution [16, 17]. As a result, an effective approach for improving the biomechanical properties of fertilizers would be to use bio-coating materials derived from natural environment-friendly and sustainable biomass sources such as lignin [18, 19], starch [10, 20], and cellulose [21, 22]. However, their downside is that these substances possess hydrophilicity and have a high cost; the resultant CRUs have poor water resistance and short nutrient-release longevity, which greatly limit their commercial applications [17, 23, 24]. It is thus important to develop new, eco-friendly, biobased coating materials with hydrophobic surfaces for controlled-release fertilizers to synchronize the nutrient release with the crops’ nutrient requirements during the whole growth period [25]. Herein, we develop environmentally friendly bio-based coating materials using biomass materials to form a hydrophobic layer outside the membrane shell by organosilicon modification [26].

Eggplant is one of the world’s top ten vegetables [27]. The global annual yield of eggplant crops increased from 3.33 billion g/ha in 2019 to 3.38 billion g/ha in 2021 [28]. The disposal of eggplant branches (EBs) after harvest has always been a serious problem globally. At present, EBs are still inseparable from the traditional treatment methods, including direct incineration, composting, and fermentation, which has caused some environmental problems such as greenhouse gas emissions [29], so the resource utilization technology of EB still needs to be further improved. Eggplant branches contain natural high-weight molecular polymers such as lignin, hemicellulose, and cellulose. They are a wasted resource with a wide range of sources [30]. Therefore, it would be of great significance and potential to use the abundant and low-cost EB as the bio-based coating raw material of CRUs. The study using EB as material has not been discovered, none of the studies attempt to liquefy EB to make coating materials. Moreover, the combination of liquefied eggplant straw and organic silicon further enhances the coating effect.

In this study, a polyurethane polymer was derived from a liquefied eggplant branch (LEB) as the coating material for CRU. Ethylene glycol (EG) and polyethylene glycol (PEG) were used as the liquefaction material [31, 32]. On the one hand, EG and PEG are often used as synthetic crosslinking agents [33]. On the other hand, when EG is heated by concentrated sulfuric acid, intermolecular water loss occurs, forming cyclic ethers and increasing the crosslinking degree. However, different liquefaction proportions (EG/PEG) will affect the content of hydrophilic hydroxyl groups in liquefaction products [34, 35], thereby affecting the coating performance. Furthermore, bio-based coatings often have many micro-holes and hydrophilic groups, because of their loose structure and the release of gases during their synthesis [35]. Therefore, the hydrophobic surface formation technology corresponds very well to CRU technology [36, 37]. The modifier, dimethyl siloxane (DS), is hydrophobic and can slow the process for moisture to enter the inner part of the CRUs through pin holes of the coating shell [36].

The objective of this study was to synthesize and evaluate the bio-based DS-modified polymer-coated urea (SCRU). First, EB was liquefied to produce bio-based coating material. Second, bio-based polyurethane was synthesized using LEB and diphenylmethane diisocyanate (MDI) to coat urea fertilizer prills. Finally, SCRU was prepared using DS to modify the bio-based polyurethane to coat the urea prills. The relationships among the N release characteristics and the coating contents were investigated. This work creates a polyurethane curing agent from bio-based coating material that has the advantages of low cost, simple preparation process, and environmental friendliness and has great potential in the large-scale production of slow-release urea, which should allow the SCRU a promising future application in agriculture production.

## Experimental

### Materials

Eggplant branch (EB), collected from Horticultural Experimental Station in Nan’jing, Jiangsu, China, was passed through a 60-mesh screen after being ground and dried in the baking oven at 105 °C for 24 h, then. Ethylene glycol (EG) was supplied by Sinopharm Chemical Reagent Co., Ltd. (Shanghai, China). Polyethylene glycol (PEG) was obatined by Sinopharm Chemical Reagent Co., Ltd. (Shanghai, China). Concentrated sulfuric acid (H_2_SO_4_, 97%) was purchased from Jinling Institute of Science and Technology (Jiangsu, China). Urea (Urea, U, particle size 3–5 mm, 46% N) was obtained by Shandong Hualu Hengsheng chemical plant. (Shandong, China). Diphenylmethane diisocyanate (MDI), was supplied by Guangzhou Hongna Chemical Co., Ltd. (Guangdong, China). Dimethyl siloxane (DS) was obtained from Tianjin Kaitong Chemical Co., Ltd. (Tianjin, China). N, N'-methylene bisacrylamide (MBA, analytical pure) was obtained by Tianjin Kaitong chemical plant. (Tianjin, China).

### Instruments

A traditional Chinese medicine powder mill (LG-01) was obtained from Zhejiang Ruian Baixin Pharmaceutical Machinery Co., Ltd. (Zhejiang, China). A collector-type constant temperature heating magnetic stirrer and an oil bath pot (DF-101S) were provided by Shanghai Yushen Instrument Co., Ltd. (Shanghai, China). A cantilever electric mixer (LC-ES-60SH) was supplied by Shanghai Lichen Bangxi Instrument Technology Co., Ltd. (Shanghai, China). The coater machine (WKY-300) was obtained by Shandong Jingcheng Pharmaceutical Equipment Manufacturing Co., Ltd. (Shandong, China). An electric heating constant-temperature blast drying oven (DGH-907385-III) was supplied by Shanghai Xinmiao Medical Device Manufacturing Co., Ltd. (Shanghai, China).

### Preparation of liquefied eggplant branch (LEBs)

**Scheme 1:**
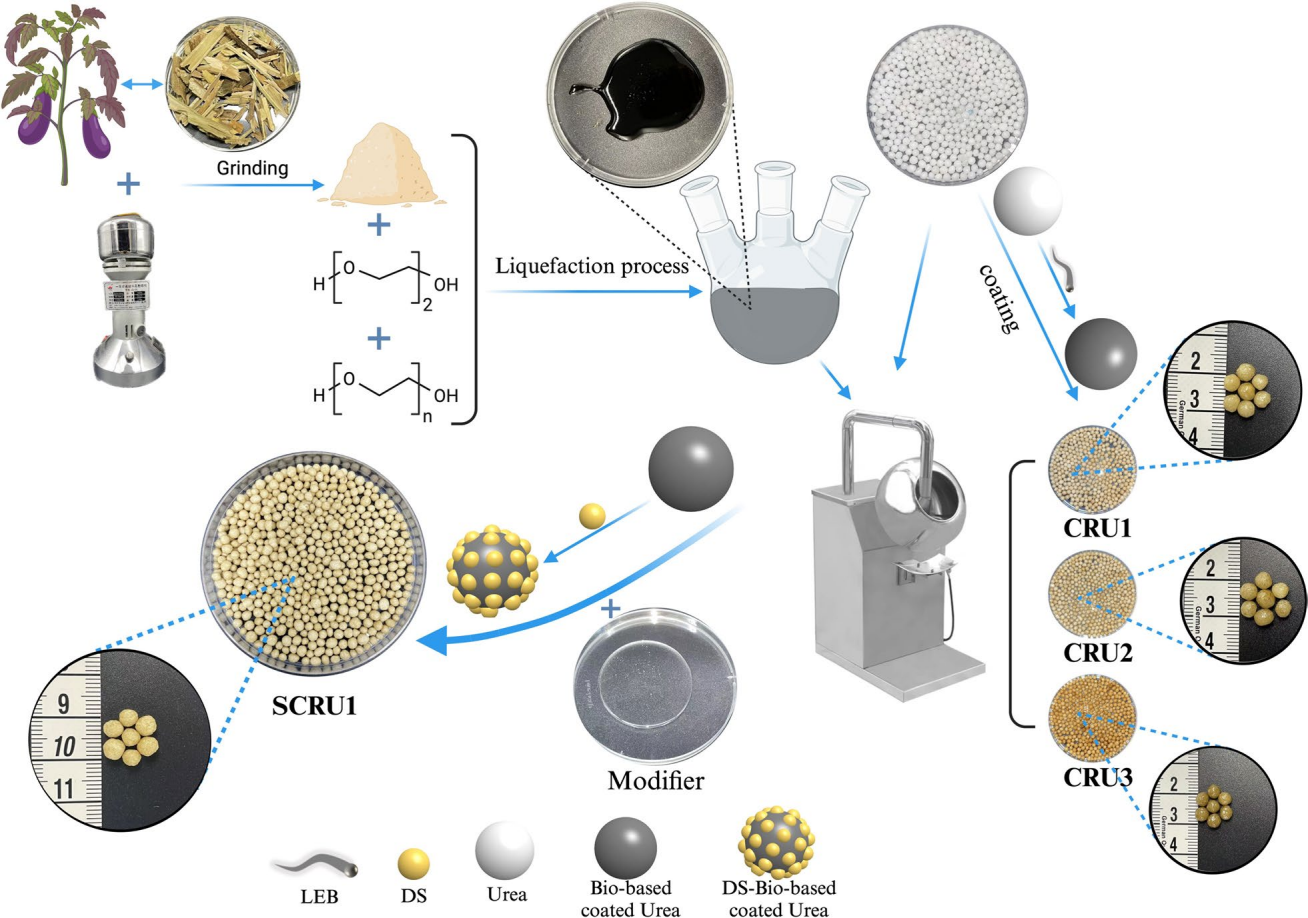
Schematic diagram of the LEB preparation process

The liquefied products were prepared in a reaction kettle, which was equipped with a mechanical stirrer, a three-necked flask, and a temperature controller. Generally, 200 mL of the three different liquefiers, namely (1) EG (120 mL) and PEG (80 mL), (2) EG (110 mL) and PEG (90 mL), and (3) EG (100 mL) and PEG (100 mL) respectively, was poured into the three-necked flask. After the temperature reached 100 °C, set the rotation speed of the mechanical stirrer at 800 rpm. Meanwhile, 50 g of dried EB powder was added to the flask with the solution and consecutive stirred until the temperature rose to 130 °C. Then, the sulfuric acid (5.4 mL) was put into the reaction kettle. The mixture was allowed to react for 1 h under atmospheric pressure at 150 °C to produce LEB1, LEB2, and LEB3, respectively [38]. After the reaction, on cooling to room temperature, the product was added to a sealed glass bottle. The above reactions were carried out under standard atmospheric pressure (101.325 kPa). The reaction can be seen in Scheme 1.

### Preparation of controlled-release urea (CRU)

Each CRU with LEB-based coating was prepared from 1000 g of urea prills (particle size 3–5 mm, 46% N). First, add the prepared urea prills and raise the temperature of the rotating drum in the coating machine to 70 ± 2 °C, then 6.66 g of MDI and 3.33 g of LEB (the 10 g of coating materials account for 1 wt% of the urea particles) were used to form bio-based coating materials [38], which were uniformly sprayed onto the surfaces of urea prills, and the thermal-curing process of the mixed coating materials was finished in the rotating drum in 15 min by hot air flow from the coater machine, and the bio-based polyurethane coating was then synthesized and attached to the surface of the urea prills. To obtain coated fertilizer with 6 wt% coating, the coating process was repeated 6 times using different LEBs (LEB1, LEB2, and LEB3) to produce CRUs (CRU1, CRU2, and CRU3), respectively.

### Preparation of dimethyl siloxane based controlled-release urea (SCRU1)

The 1000 g of urea prills prepared above were loaded into a heated rotating drum machine to 75 ± 3 °C and preheated for 30 min. Ten grams of the mixed coating material composed of 2 g of DS, 5.33 g of MDI, 2.67 g of LEB, and 0.1 g of MBA (the 10 g of coating materials account for 1 wt% of the urea particles), which was sprayed on the rotating urea particles surface, while continuing to roll the drum until the bio-based coating was completely cured [36]. The weight of bio-based coating accounted for approximately 1 wt% of that of the urea fertilizer. Similarly, to obtain coated fertilizer with 6 wt% coating, the SCRU1 was produced with the bio-based coating material by repeating the coating process 6 times.

### Characterization of LEBs, CRUs, and SCRU1

To find the liquefaction yield of LEBs (LY), 1 g of LEB (m_0_) was dissolved in 10 mL of dioxane solution (dioxane: water = 4:1 v/v), filtered, and rinsed repeatedly until colorless, then the residue (m_1_) was dried at 105 °C. The liquefaction yield was calculated using Eq. 1.

1 $$LY (\%) = 1- (m1/m0) \times 100\%$$

The surface micro-topography of the CRU1, CRU2, CRU3, and SCRU1 were analyzed by scanning electron microscope (SEM, SU8020, Hitachi, Tokyo, Japan; accelerating voltage 5 kV) under high vacuum after gold spraying on the surface of the coating. The coated fertilizers were frozen in liquid nitrogen for half an hour and then sliced with a surgical blade, and the cross sections were observed using SEM. The surface elemental compositions and distribution were determined by an energy dispersive X-ray spectroscopy (EDS, HORIBA EMAX mics2, Hitachi, Tokyo, Japan) detector attached to an SEM. The CRUs and SCRU1 were added to deionized water. After the urea of the CRUs and SCRU1 were fully dissolved, the remaining surface mask shell was dried at 60 ± 3 °C for 5 h. Meanwhile, compressed to make pellets of dried EB, LEBs, DS, CRUs, and SCRUs for Fourier transform infrared spectroscopy (FTIR, Nicolet 6700, Thermo Nicolet Corporation, Madison, Wisconsin, USA) characterization at the wavenumber range from 400 to 4000 cm^−1^ with resolution ratio 4 cm^−1^ and scanning 32 times. The coated fertilizers were crushed and rinsed with water to remove the urea, then the thermal stability of coating shells was evaluated by thermogravimetric analysis and differential scanning calorimetry (TG-DSC, Mettler TG-DSC 3 +, Netzsch, Bavaria, Germany). The sample inlet temperature was increased from 25 °C to 600 °C at a heating rate of 10 °C/min and held at 600 °C for 30 min. The data obtained were used to draw TG and DSC curves. Water contact angles (WCA, OCA50, Dataphysics, Germany) in all sample surfaces were tested using a contact angle meter.

### N release rate of CRU1 and SCRU1

The cumulative N release rates of CRU1 and SCRU1 were determined in water at 25 °C. Five grams of CRU1 or SCRU1 were put into 250 mL of deionized water, which was added to a 330 mL sealed bottle and incubated at 25 ± 0.5 °C with three replicates. To measure the amount of nitrogen released, 1 mL of each solution was removed and replaced by 1 mL of deionized water at 1, 3, 5, 7, 10, 14, 28, and 63 days or until the cumulative N release of fertilizers reached over 80%. The N concentration was determined using the Kjeldahl method [38]. The N release longevity of the coated fertilizers is defined as the time when the cumulative N release reached 80% of the total N [39].

## Results and discussion

### Composition, structure, and performance of different treatments on properties of LEBs

**Fig. 1:**
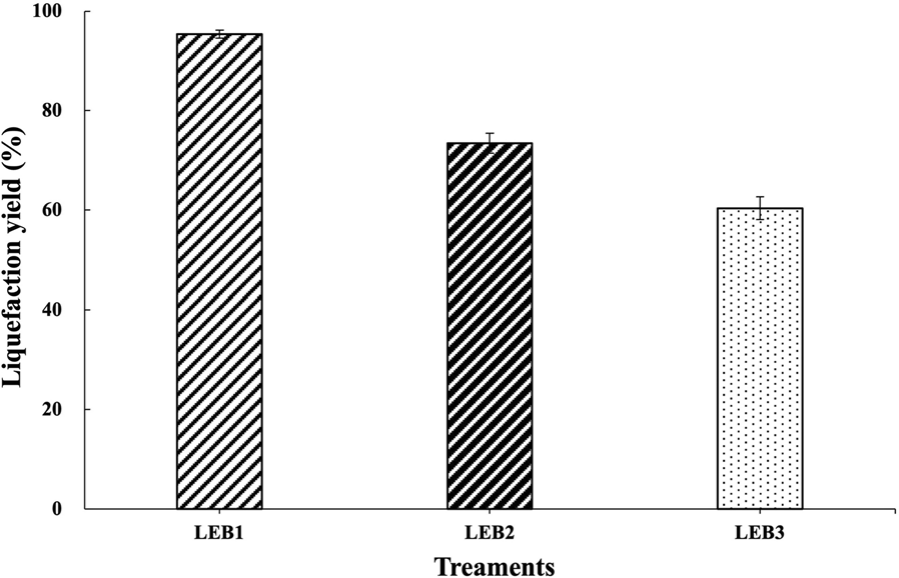
Liquefaction yields from different LEB treatments

The LY of LEB1, LEB2, and LEB3 in the eggplant branch-based liquefaction test reached 95.36%, 73.45%, and 60.37%, respectively (Fig. 1). The LY of LEB1 was as high as 95%, basically without residue, and the liquefaction yield was the highest. The result showed that LEB1 has the most market value and potential.

**Fig. 2:**
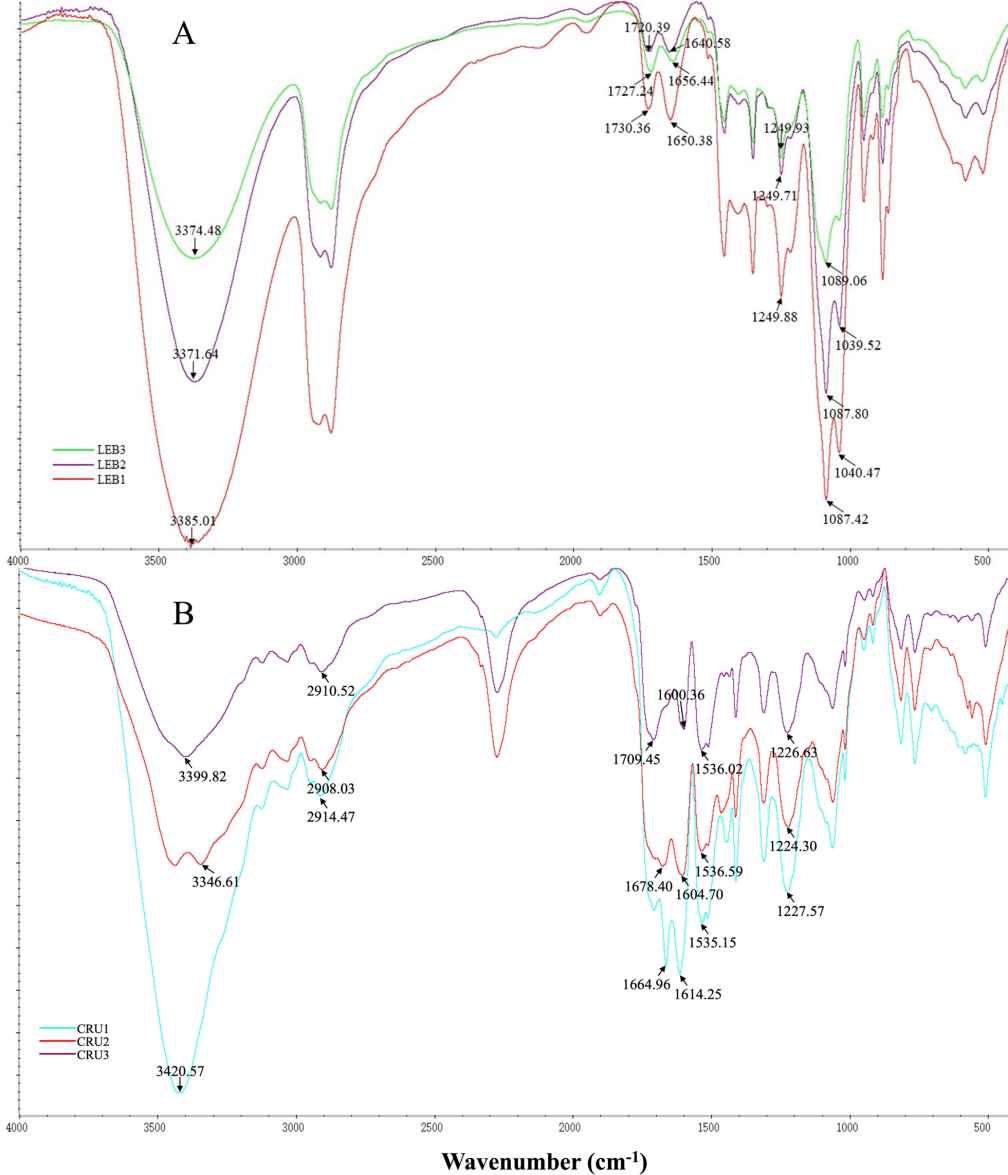
FTIR spectra of LEBs (**A**) and CRUs (**B**)

In the FTIR spectra of LEBs and CRUs showed the different chemical changes (Fig. 2). Of the LEBs, the peak intensity of LEB1 was the highest. Absorption peaks that represent the vibrations of three characteristic bonds of cellulose were observed: –OH bonds at 3385 cm^−1^, C=C of the aromatic ring at 1650 cm^−1^, and C–O–C bonds of the aromatic ring at 1249 cm^−1^, and C–O related stretching vibration absorption peaks at 1087 and 1040 cm^−1^ (LEB1 in Fig. 2A) [40]. The spectrogram of CRU shell (Fig. 2B) at 1227 cm^−1^ present the stretching vibration of C–O–C bond; the characteristic peaks of *β*N–H bonds were observed at 1535 and 1614 cm^−1^; and the characteristic peaks of N–H stretched at 3420 cm^−1^. The results confirmed that polyurethane was created by the reaction between LEB and MDI. The characteristic peak of the CRU1 shell (CRU1 in Fig. 2B), a peak at 1665 cm^−1^, corresponded to the C=C stretching of the aromatic ring. According to the peak intensity, the results confirmed that LEB1 and CRU1 was the optimal liquefied products, and suit for further research (Table 1).

**Table 1:** The peak range of the functional group

Group	–OH	C=C	C–O–C	C–O	*β*N–H
Wavenumber (cm^−1^)	3500 to 3200	1675 to 1638	1270 to 1230	1150 to 1085	1640 to 1530

**Fig. 3:**
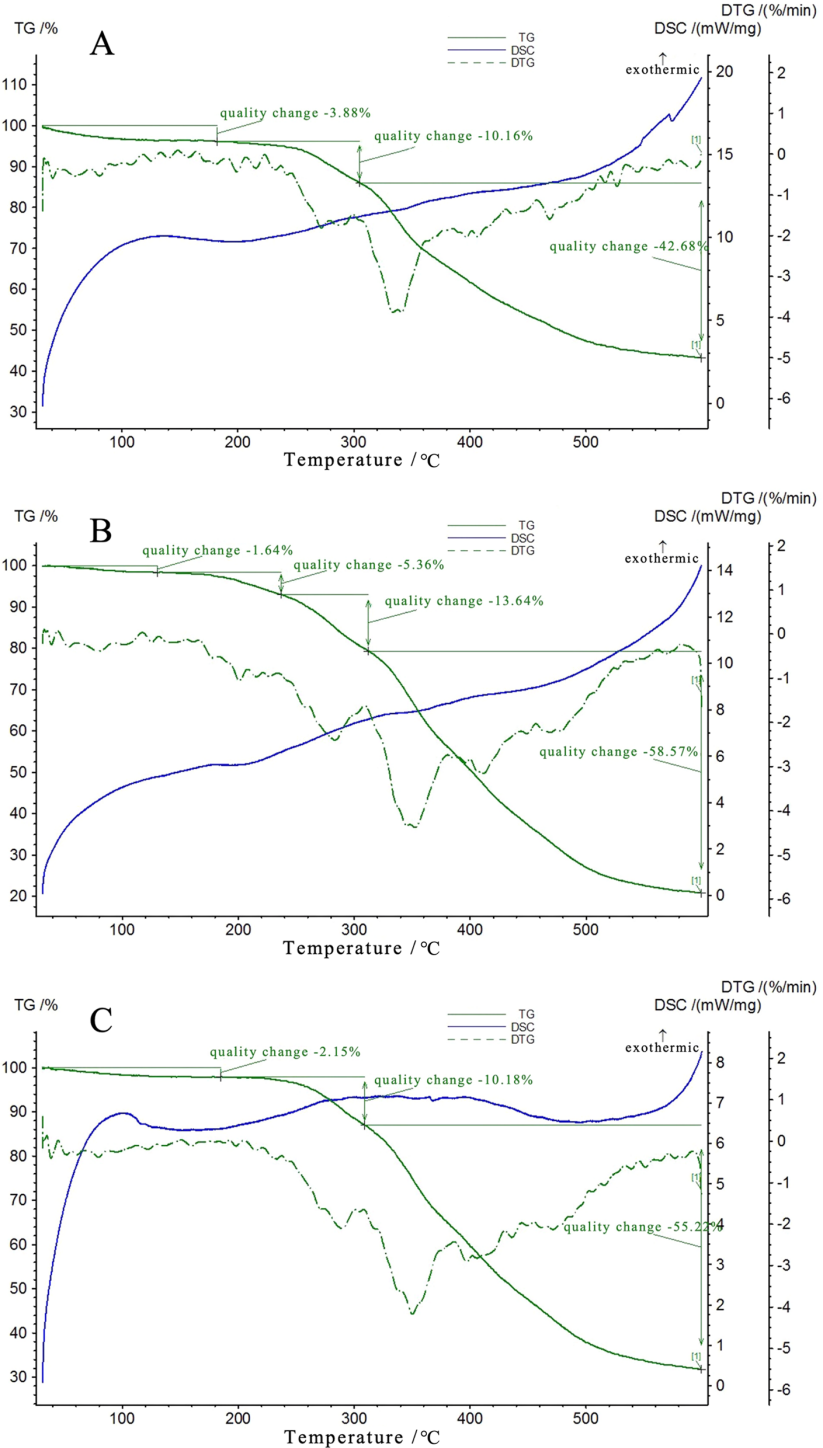
TG-DSC curve of the formed bio-based coating: **A**: CRU1, **B**: CRU2, **C**: CRU3

Thermal properties of CRU coating materials were further analyzed by TG-DSC (Fig. 3). At a certain heating rate, the pyrolysis of CRUs proceeds through several different stages as the temperature increases. The process was divided into three weight loss stages in CRUs, the first of which is in the 25–240 °C range. The initial stage of pyrolysis is generally the loss of moisture, and the CRU1, CRU2, and CRU3 weight loss rates were 3.88%, 7.00%, and 2.15%, respectively. The two main weight loss peaks of the CRUs appear between 240 and 390 °C: the hemicellulose decomposed in the range of 150–310 °C; lignin and cellulose began to degrade in the temperature range of 310–400 °C [41]. As a result, the process was mainly the decomposition of the LEB. The residual weights for CRU1, CUR2, and CRU3 were 43.28%, 20.79%, and 32.45%, respectively. In summary, compared to CRU2 and CRU3, CRU1 had minimum weight loss and the best thermal stability, which proved that LEB1 had the best performance.

### Morphology of CRUs and SCRU1

**Fig. 4:**
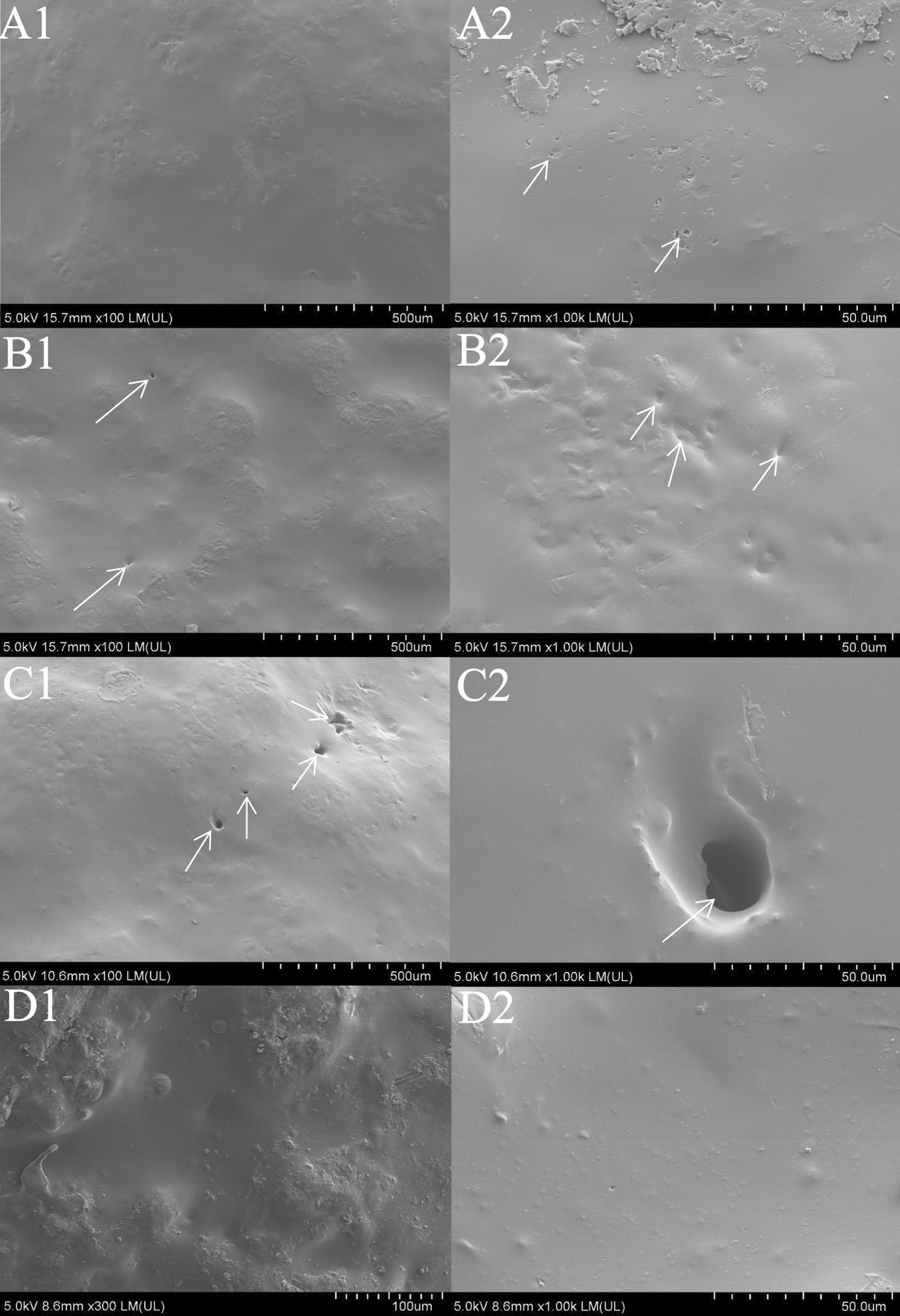
The SEM images of fertilizer surface with different coating materials CRU1 (**A1** and **A2**), CRU2 (**B1** and **B2**), CRU3 (**C1** and **C2**), SCRU1 (**D1** and **D2**)

**Fig. 5:**
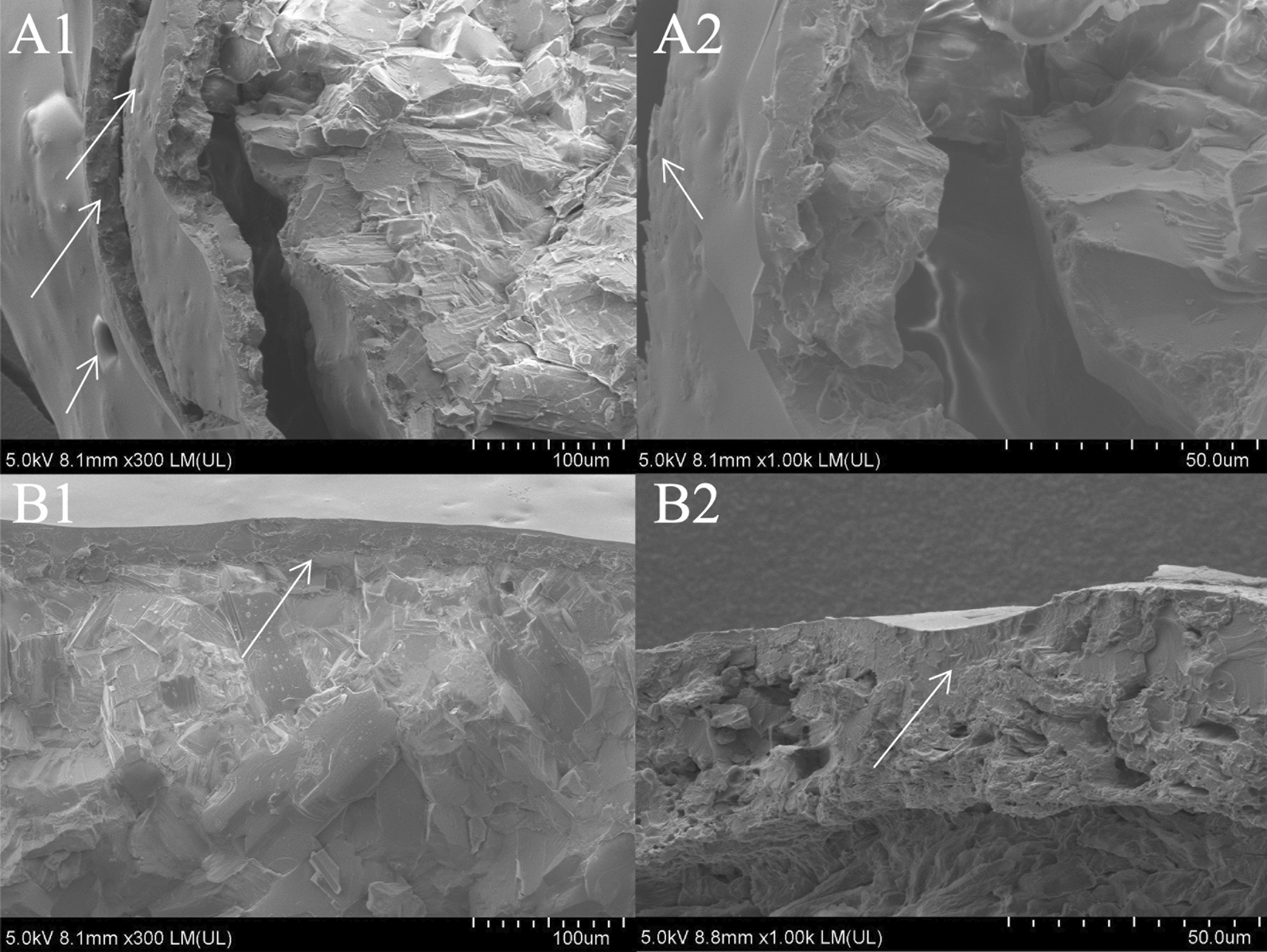
The SEM image of coating shells of CRU1 (**A1** and **A2**) and SCRU1 (**B1** and **B2**)

**Fig. 6:**
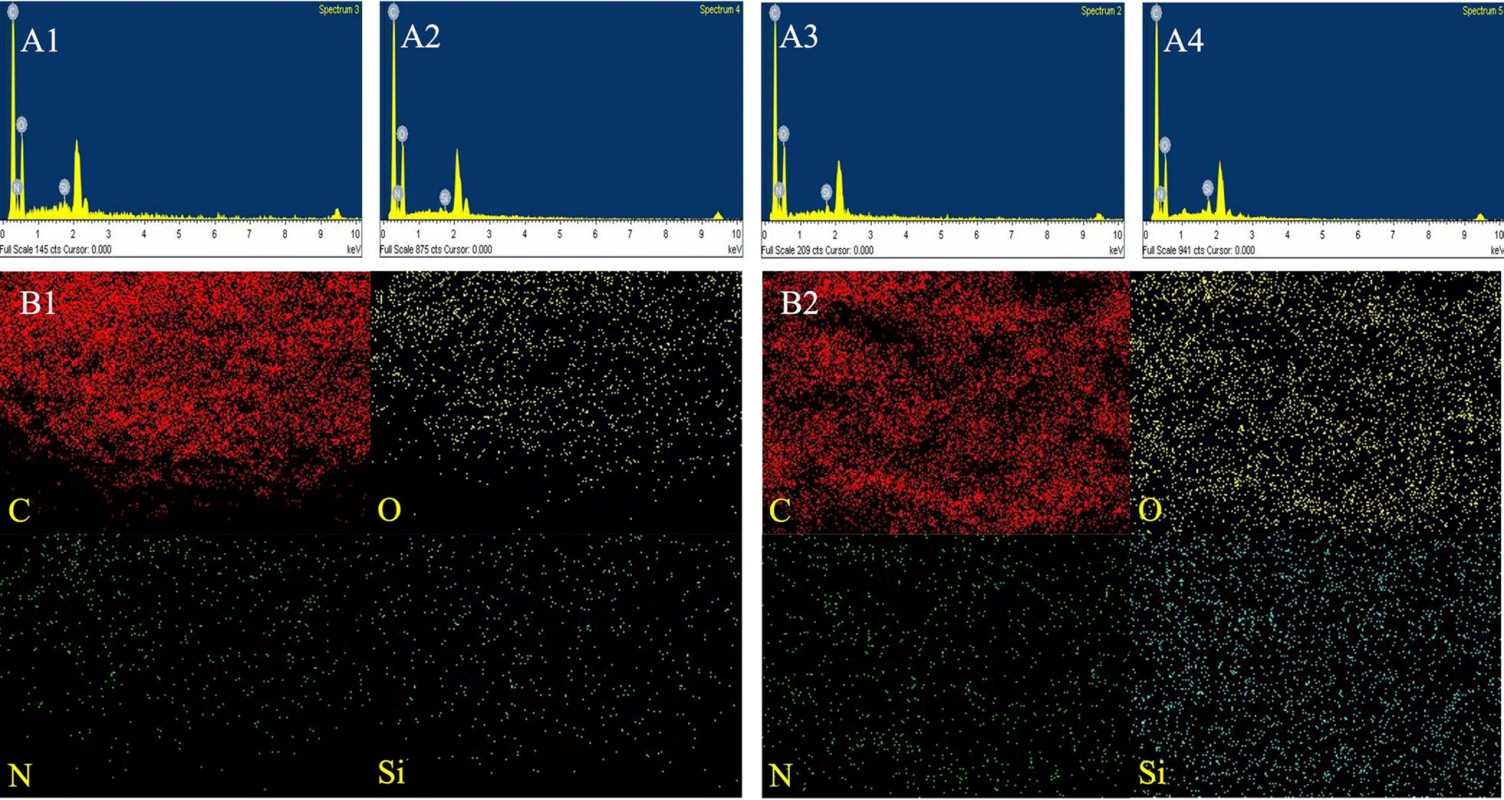
EDS spectra of CRU1 and SCRU1, and EDS maps corresponding to the SEM images for surface elemental compositions and distributions of CRU1 (**A1, A2**, and **B1**) and SCRU1 (**A3, A4**, and **B2**)

The surface morphology of CRU1, CRU2, CRU3, and SCRU1 are presented in Fig. 4. Compared with those of CRU1 (Fig. 4A1 and A2), the surfaces of the coating shells of the CRU2 (Fig. 4B1 and B2) and CRU3 membranes (Fig. 4C1 and C2) have more granular protrusions, accompanied by small pores on the surface. These micropores and an irregular structure might allow easy permeation by water, causing the quick release of nutrients from the coated fertilizer. The membrane shell of CRU1 is smoother and has fewer micropores than CRU2 and CRU3. In summary, LEB1 and CRU1 were suitable for further modification research using DS. The analyzed surface of the CRU1 without DS coating urea granules (Fig. 4A1 and A2) exhibited a rough structure with micropores rather than the surface of the DS-coated urea granules (Fig. 4D1 and D2). The result showed that the silicone atom forms a dense hydrophobic layer on the surface, which made the coating fully bonded. Furthermore, the cross-sections of the coating shells of CRU1 and SCRU1 are shown in Fig. 5. The SEM image of coating shells of the SCRU1 cross-section (Fig. 5B1 and B2) showed that the membrane shell and the fertilizer were more compact than CRU1 (Fig. 5A1 and A2), and less cracks than the coating shells of CRU1. The results also showed that the addition of organic silicon improved the crosslinking degree of LEB to a certain extent and enhanced the viscosity of LEBs. The EDS spectra and maps were obtained to determine the surface elemental compositions and contributions of CRU1 and SCRU1 (Fig. 6). The results for CRU1 (panels A1, A2, and B1 of Fig. 6) without a DS coating show that there are fewer Si elements on the surface and cross-sections of coating shell. However, after modification by DS, Si elements of SCRU1 were much more than CRU1 on the surfaces and cross-sections (panels A3, A4, and B2 of Fig. 6), and this increases the hydrophobicity of coating membrane. In addition, the C, N, O, and Si distribution on the surface of SCRU1 was comparatively uniform.

**Fig. 7:**
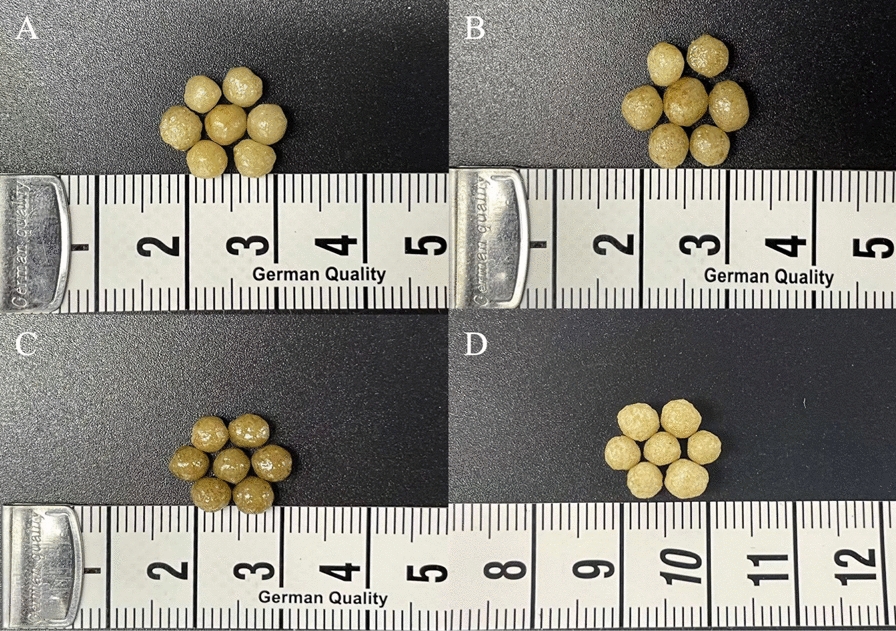
Photograph of various coated fertilizers (**A** CRU1, **B** CRU2, **C** CRU3, **D** SCRU1)

Most of the fertilizer prills were separated when the coating materials solidified on the surface of the prills. The optical image further showed a denser and smoother surface of SCRU with DS than without it (Fig. 7).

### Structural analyses of EB, LEB1, DS, CRU1, and SCRU1 by FTIR spectroscopy

**Fig. 8:**
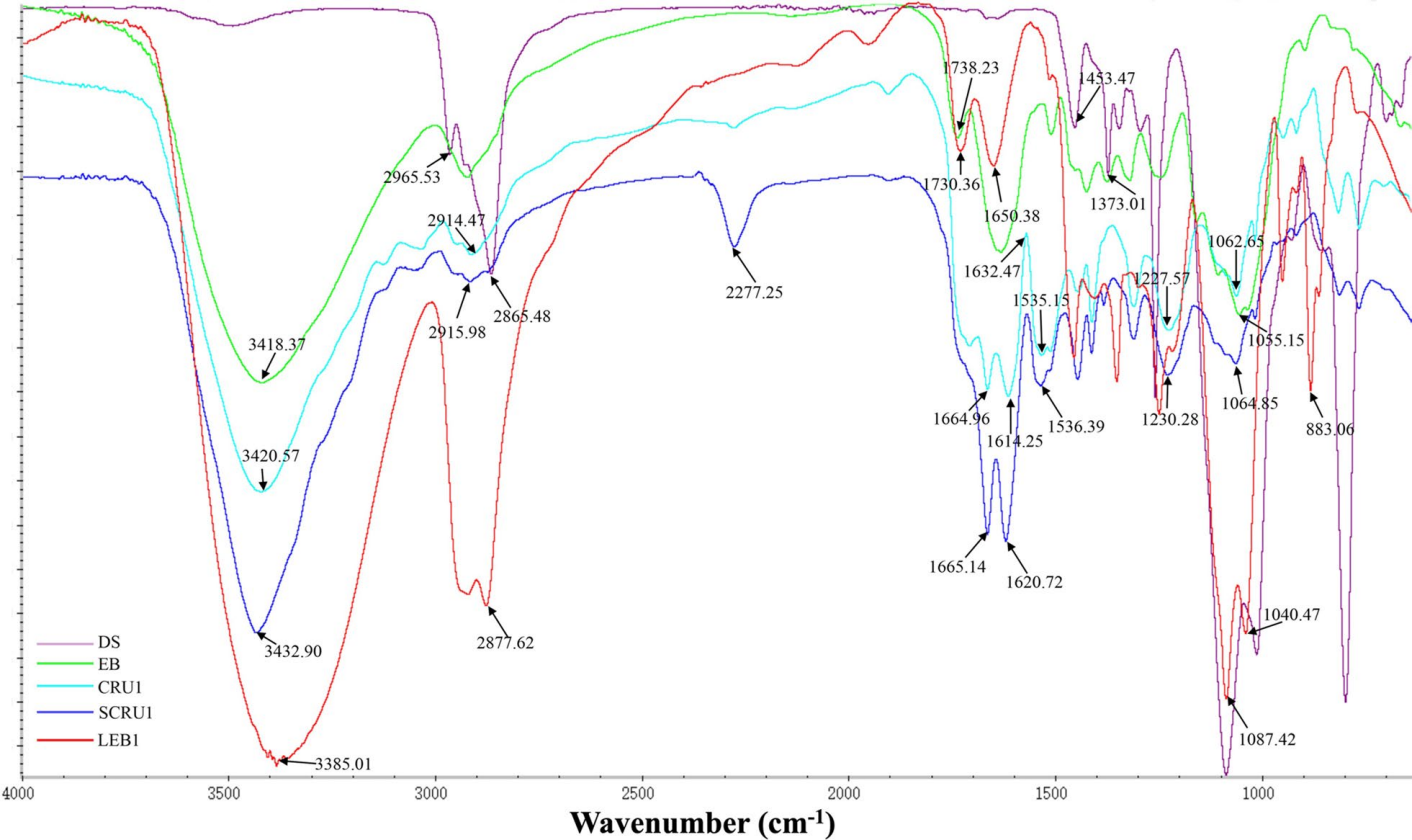
FTIR spectra of EB, LEB1, DS, CRU1, and SCRU1

The chemical shifts in the synthesis of LEBs and the coating process were revealed by FTIR spectra of EB, LEB1, CRU1, DS, and SCRU1 (Fig. 8). Three characteristic absorption peaks appear at 3418, 1738, and 1055 cm^−1^ for –OH, C=C, and C–O, respectively (EB in Fig. 8) [42]. However, in LEB1, the absorption peak corresponding to –OH shifted to the right relative to EB and appeared at 3385 cm^−1^; the characteristic peak corresponding to the C=C double bond of aromatic ring was 1650 cm^−1^, C–O–C bonds of the aromatic ring at 1249 cm^−1^, respectively. The absorption peaks of C–O bonds at 1087 cm^−1^ and 1040 cm^−1^ confirmed the existence of ternary cyclic ethers in LEB1 (LEB1 in Fig. 8).

The FITR spectra of CRU1 and SCRU1 showed that the characteristic peaks of N–H shift to the left at 3420 cm^−1^ and 3432 cm^−1^, the characteristic peaks of *β*N-H shift to the right at 1620 and 1536 cm^−1^, and showed the characteristic absorption peaks representing C–O–C bonds [43] at 1227 cm^−1^ and 1230 cm^−1^ (CRU1 and SCRU1 in Fig. 8), but the tensile vibration of SCRU1 modified by DS was more obvious than that of CRU1. These results showed that the coated fertilizer had the characteristics of EB and LEB1 at the same time. Secondly, in the wavelength ranges of 3420 cm^−1^ and 1664 cm^−1^, the absorption peaks of CRU1 and SCRU1 were consistent, which showed that the modification by DS does not destroy the original functional groups.

### TG-DSC of SCRU1

**Fig. 9:**
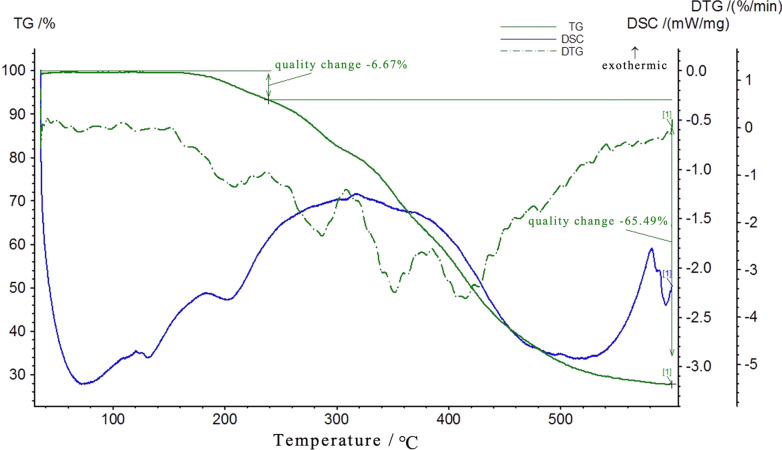
TG-DSC curve of the formed hydrophobic coating: SCRU1

Thermal properties of SCRU1 coating materials were further analyzed by TG-DSC (Fig. 9). With the increase in organosilicon content, SCRU1 had better thermal stability than CRU1. At a certain heating rate, the pyrolysis of SCRU1 and CRU1 proceeded through several different stages as the temperature increased. The process was divided into four weight-loss stages in SCRU1, the first of which is in the 100–210 °C range. The initial stage of pyrolysis is generally the loss of moisture, which mainly involves dehydration of the LEB; the SCRU1 weight loss was only 6.67%, and the CRU1 weight loss was 14.04%; thus, the thermal stability of the SCRU1 was better than that of CRU1. The two main weight-loss peaks of the CRU1 (Fig. 3) and SCRU1 appear at 240–390 °C, with a weight loss of 44% and 28%, respectively. The process was mainly the decomposition of the LEB. The final weight loss peak at 400–440 °C was mainly the decomposition of the cleavage of some residual covalent bonds in dimethyl siloxane. Most of the weight loss of CRU1 and SCRU1 occurs at 240–400 °C, and the differential value rapidly changes during this stage, which is the main stage of the thermal degradation process of the LEB.

### Surface wettability analysis of the coating material

**Fig. 10:**
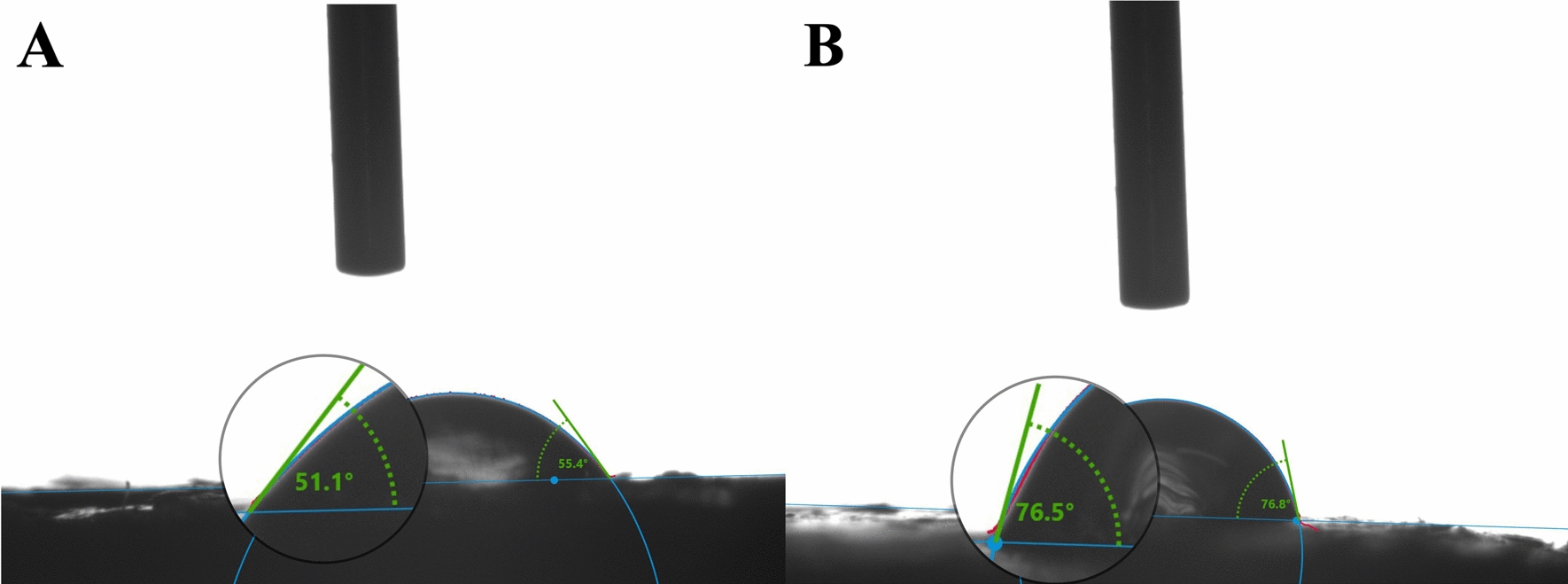
Water contact angle of CRU1 (**A**) and SCRU1 (**B**) film

The contact angle was a crucial parameter for evaluating the wettability of the material surface. As shown in Fig. 10, the surface WCA of CRU1 film (Fig. 10A) is 51.1°, When DS was added to the bio-based LEB coating material, the surface WCA of SCRU1 film (Fig. 10B) increased to 76.5°. In summary, the hydrophobicity of the surface film increased after the addition of the hydrophobic coating silicone, which was because the hydrophobic silicon atoms uniformly expanded outward after the grafting of silicone and the bio-based LEB coating chain, forming a hydrophobic barrier outside the coating material. The DS can make coating shells with hydrophobic, which is more beneficial to slowing down the release of nutrients.

### Nutrient controlled-release abilities of the CRU1 and SCRU1

**Fig. 11:**
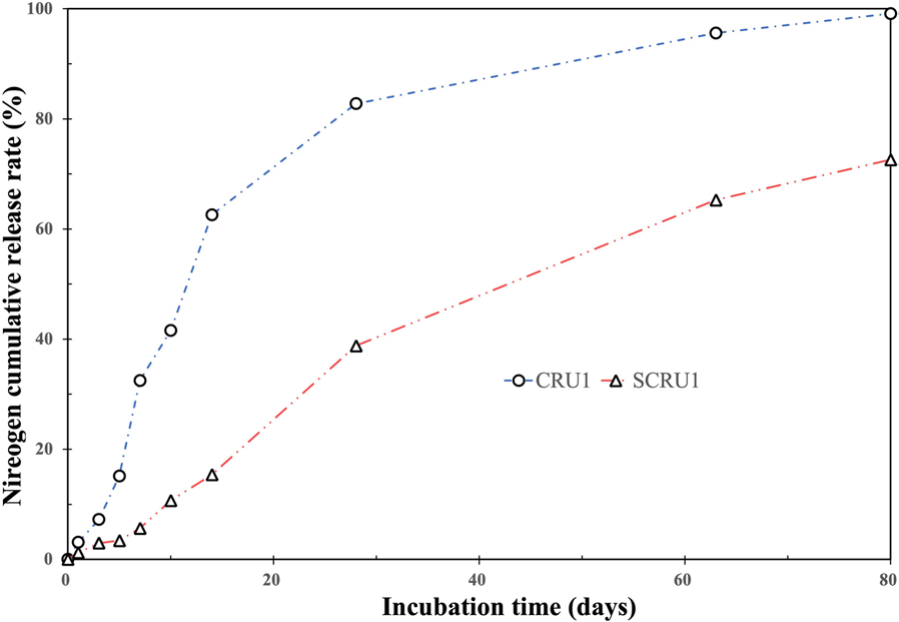
Release curves of N from fertilizers coated with different materials (6% coating content) at 25 °C in water

The experimental result indicated that, with the added DS, the nutrient release longevity increased at first and then decreased (Fig. 11). The nitrogen cumulative release rate of CRU1 reached 82.75% after 28 days, and nitrogen cumulative release rate of SCRU1 was 28.79%. The nitrogen cumulative release rate within 28 days should be less than 75% as specified by the CRU international standard (ISO 18644-2016) [44]. Therefore, the controlled-release performance of SCRU1 was the best. After 63 days, the nitrogen cumulative release rate reached 95.58% for CRU1 and 65.26% for SCRU1. On the 80th day, the nitrogen cumulative release rate was 99.12% for CRU1 and 72.59% for SCRU1. Compared to CRU1, the Si elements on the membrane shell of SCRU1 increased after adding DS (Fig. 6), and the WCA increased from 51.1° to 76.5° (Fig. 10). In summary, silicone atoms form a dense hydrophobic layer, and the controlled-release performance of fertilizers is further improved, nutrient release cycle is extended and hydrophobic performance was improved after the excellent combination of DS and LEB1.

## Conclusions

An environmentally friendly bio-based coating material was successfully fabricated to develop novel CRUs. Three different CRUs, including CRU1, CRU2, and CRU3, were fabricated and characterized, and CRU1 showed the best performance. The optimum proportion of EG/PEG to prepare LEB was 120:80. Furthermore, the hydrophobic material DS was employed to modify the bio-based controlled-release fertilizer as a cross-linking agent to increase the hydrophobicity of SCRU1, to block the pore channels, and to reduce cracks in biopolymer coating membranes. These three factors dramatically improved the nutrient release characteristics of the SCRU1. The hydrophobicity was mainly attributed to the organosilicon atoms on the SCRU1 surface that formed a hydrophobic layer, preventing external water from contacting the coating materials. This newly fabricated fertilizer has many advantages over current commercial bio-based coating materials, including being environmentally friendly, highly efficient and renewable. The findings of this study thus display a great potential for large-scale applications to satisfy the increasing demand for controlled-release ureas because the materials developed herein are cost-effective and environmentally friendly. Furthermore, it should be noted that this study was conducted under laboratory conditions, and the actual steps still needed to be done before large-scale industrial production. Such as, further optimization of relevant process conditions to improve the production efficiency of bio-based controlled-released urea, and efficient collection and storage of eggplant branches.

## Data Availability

All data generated or analyzed during this study are included in this manuscript.

## Abbreviations

CRUs: Controlled-release ureas
SCRUs: Dimethyl siloxane based controlled-release ureas
EBs: Eggplant branches
LEB: Liquefied eggplant branch
EG: Ethylene glycol
PEG: Polyethylene glycol
DS: Dimethyl siloxane
MDI: Diphenylmethane diisocyanate
MBA: N, Nʹ-methylene bisacrylamide
LY: Liquefaction yield
SEM: Scanning electron microscope
EDS: Energy dispersive X-ray spectroscopy
FTIR: Fourier transform infrared spectroscopy
TG: Thermogravimetric
DSC: Differential scanning calorimetry
WCA: Water contact angles
